# Preparation, Structural Identification, and Screening of Egg-Derived Peptides with Facilitating Alcohol Metabolism Activity

**DOI:** 10.3390/foods13050745

**Published:** 2024-02-28

**Authors:** Yali Tan, Yulin Wang, Yuan Wan, Yu Liang, Qiaocui Liu, Mengya Wei, Tao Hou

**Affiliations:** 1 Key Laboratory of Egg Processing, Ministry of Agriculture and Rural Affairs, Wuhan 430000, China; tanyali@webmail.hzau.edu.cn (Y.T.); shendanfzb@163.com (Y.W.); 2 College of Food Science and Technology, Huazhong Agricultural University, Wuhan 430070, China; wanyuan@webmail.hzau.edu.cn (Y.W.); liangyu@webmail.hzau.edu.cn (Y.L.); liuqiaocui_24@163.com (Q.L.); weimengya@webmail.hzau.edu.cn (M.W.); 3 Key Laboratory of Environment Correlative Dietology, Huazhong Agricultural University, Ministry of Education, Wuhan 430000, China

**Keywords:** anti-alcohol activity, egg, molecular docking, structure identification

## Abstract

The aim of this study was to obtain egg-derived peptides with facilitating alcohol metabolism (EPs) by enzymolysis, to identify their structures, and screen small polypeptides with higher activity by molecular docking. The optimum conditions for preparing EPs with facilitating alcohol metabolism were obtained by a single factor experiment, adding 2% Protamex and performing enzymolysis for 3 h with a liquid–material ratio of 35:1. The dose–response relationship experiment showed that 800 mg/kg·bw EPs played a better role in facilitating alcohol metabolism. EPs contained 40% hydrophobic amino acids (HAA), including 9.24% Leu. Eighty-four peptides were identified by HPLC-MS/MS and four peptides with potential activation of alcohol dehydrogenase were further selected by molecular docking. The tetrapeptide Trp-Ile-Val-Asp (WIVD) with the highest binding energy reached −7.16 kcal/mol. These findings suggest that egg is a good source for the preparation of peptides with facilitating alcohol metabolism activity.

## 1. Introduction

At present, the worldwide consumption of all kinds of alcohol and alcoholic beverages is increasing [1,2]. Moderate drinking can promote digestion, eliminate fatigue, stabilize blood sugar, and improve sleep quality [3]. However, excessive drinking can lead to alcoholism, causing headaches, dizziness, vomiting, and other uncomfortable symptoms. Long-term drinking can also cause damage to the central nervous system and digestive system, leading to fatty liver, cirrhosis, and other diseases [4,5].

After drinking alcohol, alcohol begins to be absorbed in the mucous membrane of the digestive tract, enters the blood through the capillaries of the stomach and small intestine, and then enters the organs of the human body through blood circulation [6]. About 90% of alcohol is metabolized in the liver, and the remaining 10% is excreted through breath, sweat, and urine [7]. In the liver, there are three systems that metabolize alcohol, which are in three different cellular compartments, as follows: the alcohol dehydrogenase system (ADH) in the cytoplasm, the microsomal alcohol oxidation system (MEOS) in the endoplasmic reticulum, and the catalase system (CAT) in the peroxidase [8]. Among these, ADH and MEOS are the main pathways of ethanol metabolism [9]. When alcohol is ingested, it is metabolized primarily through the ADH system [10]. Under the catalysis of alcohol dehydrogenase and oxidized coenzyme I (NAD^+^), alcohol is directly oxidized to acetaldehyde [11]. This process also converts NAD^+^ into reduced coenzyme 1 (NADH + H^+^), increasing its accumulation in the liver, and in turn producing cytotoxicity and leading to liver cell damage [12]. On the other hand, alcohol metabolism leads to the production of reactive oxygen species (ROS), including O^2−^, ·OH, and H_2_O_2_, NO, O_3_. [13]. These free radicals not only promote cell oxidation and the release of inflammatory factors, but also rapidly combine with alcohol molecules to form active metabolites, such as hydroxyl free radicals and hydroxyethyl free radicals, which cause excessive oxidation of lipid on the surface of liver cells, and thus hinder liver cell metabolism of fatty acids [9,14].

In recent years, many studies have been carried out on food-borne biomolecules with anti-alcohol effects [15,16]. Currently, these biomolecules can be roughly divided into the following four categories: polyphenols, polysaccharides, peptides, and microbial anti-alcohol metabolites [17,18]. Peptides have a smaller molecular weight (MW) and are therefore more easily absorbed by the body in order to function [19]. A previous study found that black soybean-derived peptides showed strong protective effects against alcohol-induced liver injury in mice [20]. Compared with the model group, the levels of alanine aminotransferase (ALT), aspartate aminotransferase (AST), triglyceride (TG) and creatinine (Cr) in serum were significantly decreased, and pathological changes in liver tissue were significantly alleviated after treatments with black soybean-derived peptides. It is speculated that the potential mechanism of black soybean-derived peptides in alleviating alcoholic liver injury in mice may be related to their effective antioxidant activity (CAT, GSH, SOD, and MDA) and anti-inflammatory properties (IL-1β and IL-6). Additionally, there is a health care product called Haiwangjinzun (HWJZ) on the market, which is mainly made of oyster soy peptides, vitamin c, L-cysteine, taurine, and other raw materials. It has a protective effect against chemical liver injury and has a significant prevention and treatment effect on alcoholism, reducing the degree of drunkenness and promoting alcohol metabolism [21,22]. It is one of the few products on the market with a health food batch number to promote alcohol metabolism.

Eggs are rich in protein, lecithin, vitamins, calcium, iron, phosphorus, and other trace elements, contain eight essential amino acids (EAA), and the composition of human protein is very similar [23]. Because of their high nutritional values, easy availability of raw materials and low price, they have become a good source for developing bioactive peptides in recent years [24,25]. To date, bioactive peptides with antioxidant [26], antihypertensive [27], antibacterial [28], anti-inflammatory [29] and other effects have been isolated and extracted from eggs [30,31]. However, there are no reports on the preparation of accelerating alcohol metabolism peptides from eggs. Thus, the purpose of this study is to prepare egg-derived peptides with facilitating alcohol metabolism activity, to explore the optimum preparation technology and the dose–response relationship. More importantly, the structures will also be identified and the vital structures promoting alcohol metabolism will be elucidated.

## 2. Materials and Methods

### 2.1. Materials and Reagents

Fresh eggs were purchased from Hubei Shendan Health Food Co. (Xiaogan, China). Alcalase, Neutrase, Protamex, papain and trypsin were purchased from Novozymes, Denmark. HPLC grade alcohol and n-butanol were purchased from Comet and Aladdin. All other chemical reagents are analytically pure and were purchased from Sinopharm Chemical Reagent Co. Commercial kits for determining the activities of serum ALT, AST, and hepatic reduced glutathione (GSH) were purchased from Jiancheng Bioengineering Institute (Nanjing, China). Liver alcohol dehydrogenase (ADH), acetaldehyde dehydrogenase (ALDH), and malondialdehyde (MDA) activity assay kits were purchased from Suzhou Comin Biotechnology Co. (Suzhou, China).

### 2.2. Preparation of Egg-Derived Peptides with Facilitating Alcohol Metabolism (EPs)

Firstly, the distilled water was mixed with the whole egg and then treated in a boiling water bath for 30 min. Secondly, the pH was adjusted to the optimal pH of the enzyme used with 0.5 M NaOH, and then the temperature was adjusted to the optimal temperature of the enzyme used (see Section 2.3). The appropriate amount of enzyme was added to start the enzymatic digestion. The pH was kept constant with 0.5 M NaOH during the enzymatic digestion process. Finally, the obtained hydrolysate was boiled in a water bath for 10 min to inactivate the enzyme. The enzymatic hydrolysate was centrifuged at 5000× *g* for 15 min at 4 °C, then the supernatant was collected and lyophilized for the further studies.

### 2.3. Optimization of Enzymatic Hydrolysis Conditions

Under the conditions of a liquid–material ratio of 20:1, enzyme additive quantities of 3% (*w*/*w*), enzymatic hydrolysis time of 2 h, optimal temperature and pH of each protease, the effects of Alcalase (pH 8.0, 55 °C), Neutrase (pH 7.0, 50 °C), Protamex (pH 7.0, 55 °C), papain (pH 6.5, 50 °C), and trypsin (pH 7.5, 37 °C) on the enzyme digestion of whole egg were investigated, and the hydrolysis degree (DH), blood alcohol concentration (BAC) elimination rate in vivo, and ADH activation rate in vitro were used as the indexes to screen out the optimal enzyme.

The optimal protease, the DH and BAC elimination rate in vivo and the ADH activation rate in vitro were used as the indicators to determine the optimal liquid-material ratios (*v*/*w*) (20:1, 25:1, 30:1, 35:1, and 40:1), the enzyme additive quantity (*w*/*w*) (1%, 2%, 3%, 4%, and 5%), and the optimal enzymatic hydrolysis time (1 h, 2 h, 3 h, 4 h, and 5 h).

### 2.4. Determination of the DH

The determination of DH was calculated by the pH-state method [32]. DH is calculated as follows:

DH (%) = B × N × α^−1^ × M^−1^ × h_tot_^−1^ × 100% (1)

where B-amount of NaOH (L); N-molar concentration of NaOH = 0.5 M; α-average degree of dissociation of α-α-NH_2_ group = 1.01; M-mass of protein (g); h_tot_-total number of peptide bonds of the protein substrate, 7.6 mmol/g for egg protein [33].

### 2.5. Experimental Animals and Treatments

Kunming male mice (23–27 g, 4 weeks) were obtained from Hubei Laboratory Animal Research Center (Hubei, China). All the procedures performed were according to the Guidelines for Care and Use of Laboratory Animals of Huazhong Agricultural University, and the animal ethics approval number was HZAUMO-2023-0287.

After one week of adaptation, the mice were randomly divided into 6 groups with 10 mice in each group, namely the model group and different protease treatment groups, different liquid–material ratios, different enzyme additive quantity, or different enzymatic hydrolysis time groups. Animals were fasted for 12 h before the experiment but were allowed access to water. All groups were given 53% alcohol (*v*/*v*) (10 mL/kg·bw) and then given 400 mg/kg·bw of EPs after 15 min (the model group was given the corresponding dose of saline). Blood was collected after 35 min. The blood samples were centrifuged at 4 °C and 3000× *g* for 10 min, and the serum was stored in a refrigerator at −80 °C for further studies [34].

The dose–response relationship experiment of egg-derived peptide with the highest facilitating alcohol metabolism activity was prepared by the optimum process according to the following steps. HWJZ was selected as a positive control [35]. Kunming male mice (25 ± 2 g) were randomly divided into 8 groups with 10 mice in each group after adaptation for a week, as follows: Normal group, model group, positive control HWJZ group (7.5 mL/kg·bw), 200 mg/kg·bw, 400 mg/kg·bw, 600 mg/kg·bw, 800 mg/kg·bw, 1000 mg/kg·bw EPs group. The normal group was given 10 mL/kg·bw physiological saline, and the other groups were given 53% alcohol (*v*/*v*) (10 mL/kg·bw). After 15 min, the HWJZ and different doses of EPs (normal group and model group were given 10 mL/kg·bw physiological saline) were given. The mice were killed 35 min later, and blood and liver samples were collected. The blood was centrifuged at 4 °C and 3000× *g* for 10 min, the serum was removed, and the liver was refrigerated at −80 ℃ for further analysis of biochemical indicators.

### 2.6. Determination of the BAC by Gas Chromatography (GC)

The instrument conditions were as follows: Gas chromatograph (GC2010 Plus), TG-WAXMS capillary column: 30.0 m × 0.25 mm × 0.25 μm, inlet temperature 150 °C, FID detector temperature 250 °C, sample size 1 μL, programmed temperature rise: 40 °C (5 °C/min) −60 °C (20 °C/min) −120 °C, and retention time 3 min. Alcohol (chromatographic grade) standard solutions of 0.1%, 0.2%, 0.4%, 0.6%, 0.8%, 1.0%, 1.5%, and 2.0% (*v*/*v*) were prepared. The alcohol standard solution of different concentrations was mixed with the 0.05% n-butanol internal standard at a 1:1 volume ratio. The peak area ratio of alcohol to n-butanol was used as the standard curve for the concentration of ethanol standard solution. Mouse serum were mixed with 0.05% n-butanol (*v*/*v*) at a 1:1 volume ratio, and then measured on the machine. Peak areas of alcohol and n-butanol were recorded, and the concentration of alcohol in the sample was calculated through the standard curve, which showed the concentration of blood alcohol in the sample. The BAC elimination rate is calculated as follows:

(2) $$\mathrm{BAC}\;\mathrm{elimination}\;\mathrm{rate}\;(\%)=\frac{Average\;BAC\;in\;model\;group-BAC\;in\;given\;EPs\;group}{Average\;BAC\;in\;model\;group}\;\times 100\%$$

### 2.7. Determination of Biochemical Indicators

The serum AST and ALT levels of the mice and the ADH, ALDH, GSH, and MDA of the mice livers were measured using commercial kits, according to manufacturers’ instructions.

### 2.8. Determination of the ADH Activation In Vitro

ADH activity was determined using the Valle & Hoch method with slight modifications. Specifically, 1.5 mL of sodium pyrophosphate buffer with pH 8.8 was added to 1.0 mL of 27 mM oxidized coenzyme I (NAD^+^), 0.5 mL of 11.5% alcohol (*v*/*v*), and 0.1 mL of sample solution in a test tube, mixed, and then water-bathed for 5 min at 25 °C. Immediately after the water bath, 0.1 mL 0.25 U/mL ADH was added and the absorbance value at 340 nm was measured by spectrophotometer. Readings were taken at 10 s intervals for 5 min, and the initial linear portion of the reaction was taken for graphing. The 0.5 mL distilled water component was substituted for 0.5 mL of 11.5% alcohol as a reference zero setting. The 0.1 mL distilled water component was substituted for 0.1 mL EPs to determine the absorbance of the control group. The slope of the EPs group was recorded as Vs, and the slope of the non-EPs group was recorded as Vo. The ADH activation rate is calculated as follows:

(3) $$\mathrm{ADH}\;\mathrm{activation}\;(\%)=\frac{Vs-Vo}{Vo}\;\times 100\%$$

### 2.9. Amino Acid Analysis

According to the national standard of the People’s Republic of China GB5009.124-2016, the amino acids composition in EPs was determined by ninhydrin post-column derivatization [20]. Amino acid analysis was performed on a Hitachi L-8900 Amino Acid Analyzer (Hitachi, Tokyo, Japan) (Column: sulfonic acid type cationic resin; Detection wavelength: 570 nm and 440 nm). The principle is to hydrolyze EPs with HCl to obtain free amino acids, which were separated by ion exchange column to produce a color reaction with ninhydrin solution. Specifically, 30 mg of EPs and 10 mL of 6 M HCl were added to the hydrolysis tube. It was hydrolyzed under vacuum at 110 °C for 22 h and then dried. Then, an appropriate amount of sodium citrate buffer was added to dissolve, through a 0.22 μm filter membrane, and the filtrate was collected as the sample determination solution. Mixed amino acid standard working solution (100 nmol/mL) was used as an external standard, comprising 16 kinds of amino acids. The mixed amino acid standard working solution and the sample determination solution were injected into the amino acid analyzer in the same volume, and the amino acid concentration in the sample determination solution was calculated by the peak area of the external standard method.

### 2.10. Characterizations of the Structures of EPs

The structure of EPs was identified by UPLC-ESI-MS/MS (Thermo Fisher Q Exactive Focus). EPs was dissolved in deionized water at 1 mg/mL concentration. Then it was filtered using a 0.22 μm filter membrane and the filtrate was collected as the sample solution for determination. Chromatographic conditions were as follows: Column: Accucore C-18 (2.6 μm, 100 × 2.1 mm); Mobile phase A: 0.1% formic acid-acetonitrile, B: 0.1% formic acid aqueous. Gradient elution conditions: 0–45 min, mobile phase A: 1–30%; 45–50 min, mobile phase A: 30–90%; 50–52 min, mobile phase A: 90%; 55–60 min; Mobile phase A: 90–1%. Injection concentration: 1 mg/mL; Flow rate: 0.2 mL/min; Column temperature T = 45 °C; Detection wavelength: 214/280 nm. Both formic acid and acetonitrile are Fisher reagents.

Mass spectrum conditions were as follows: ESI + MS ion source detection mode, spray voltage 3800 V, shielding gas (N_2_) pressure 40 psi, dry gas (N_2_) flow rate 10.00 L/min, capillary temperature 275 °C, mass charge ratio (*m*/*z*), scanning range 100–1000.

Thermo Flyer Proteome Discoverer 2.4 software was used to analyze the results. After the initial analysis of the peptide sequence, the resolved peptide segments were searched and matched in the UniProt database (https://www.uniprot.org/ (accessed on 10 November 2023)) to determine the structure of the peptide segment.

### 2.11. Molecular Docking and Visual Analysis

The three-dimensional structure of polypeptide was constructed in Chem Office 2020 software. The crystal structure of ADH (PDB ID: 5ENV) was downloaded from the RCSB Protein Data Bank (PDB, https://www.rcsb.org/ accessed on 20 November 2023) and PyMol 2.6.0 software was used to remove the ligand. The ADH and polypeptide were opened in Autodock Tools 1.5.6 software, and, after water removal and hydrogenation treatment, a semi-flexible docking method was adopted for docking. The docking center site was set as (−47.3, 44.4, −22.6), the docking box size was 40 Å × 40 Å × 40 Å, and the docking binding energy was recorded [36]. The five results with the lowest binding energy were selected for visual analysis. LigPlot^+^ 2.2.5 software for two-dimensional visual analysis and PyMol software for three-dimensional visual analysis were used.

### 2.12. Peptide Synthesis

Peptides (purity > 95%) were synthesized and provided by Hefei ChinaPeptide Biotechnology Co., Ltd. (Hefei, China) using Fmoc solid-phase synthesis.

### 2.13. Statistical Analysis

Statistical analysis was performed using SPSS 26.0 software (SPSS Inc, Chicago, IL, USA). Analysis of variance (ANOVA) and mean comparisons were done using Duncan’s multiple range test. *p* < 0.05 indicated statistical significance. GraphPad Prism 9.0 software was used to calculate the EC50 value. The built-in nonlinear regression dose–response model in GraphPad Prism 9 software was selected. All data were expressed as mean ± standard deviation (SD).

## 3. Results and Discussion

### 3.1. Optimization of Enzymatic Hydrolysis Conditions

**Figure 1:**
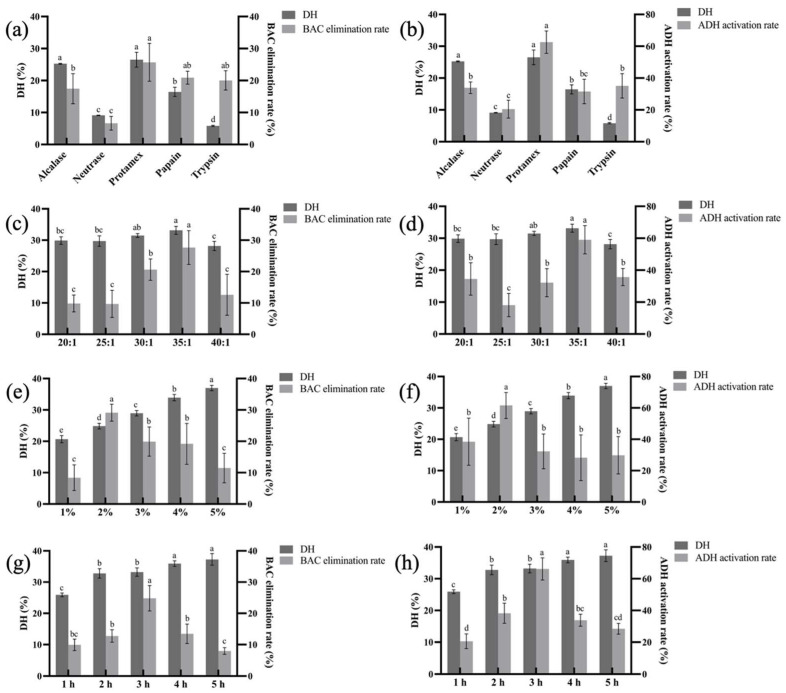
The effect of different proteases (**a**,**b**), liquid–material ratios (**c**,**d**), enzyme additive quantities (**e**,**f**), enzymatic hydrolysis times (**g**,**h**) on the DH, BAC elimination rate, and ADH activation rate. Different letters indicate significant differences (*p* < 0.05).

As shown in Figure 1a,b, the whole egg enzymatically hydrolyzed by Protamex showed the highest DH, while the BAC elimination rate in vivo and the ADH activation rate in vitro showed relatively consistent results. The BAC elimination rate of the EPs obtained by the Protamex reached 25.70% and the ADH activation rate reached 62.57%, which were better than the values obtained in other proteases (*p* < 0.05) treatment groups. Therefore, Protamex was chosen for enzymolysis of the whole egg to obtain EPs with higher facilitating alcohol metabolism activity.

Figure 1c,d shows that the DH increased with the liquid–material ratio, reaching its highest at 35:1, and then decreased. Meanwhile, the BAC elimination rate reached 27.65% at 35:1, which was significantly better than that in the other groups. The same trend was obtained in the in vitro experiment, with the highest ADH activation rate of 59.06% at 35:1 (Figure 1d).

As shown in Figure 1e,f, with the increase of enzyme additive quantity, the DH showed a trend of gradual increase. The BAC elimination rate and ADH activation rate both increased firstly and then decreased with more enzyme additions. When the amount of enzyme was 2%, the BAC elimination rate and ADH activation rate reached the peak of 29.13% and 61.54%, respectively.

As shown in Figure 1g,h, the DH increased slightly with the prolongation of enzymatic hydrolysis time. The BAC elimination rate and ADH activation rate showed a trend of elevating and reached the maximum of 24.83% and 66.18%, respectively. After three hours, the BAC elimination rate and ADH activation rate decreased with a longer treatment time. This might be due to excessive hydrolysis leading to structural changes in a portion of the peptides with facilitating alcohol metabolism activity.

Using egg as a raw material, the DH, BAC elimination rate, and ADH activation rate were selected as indexes to determine that the best protease for hydrolyzing egg was Protamex. A single factor experiment was used to optimize the preparation process of egg-derived peptide with facilitating alcohol metabolism as follows: liquid–material ratio of 35:1, enzyme additive quantity of 2%, and enzymatic hydrolysis time of 3 h.

### 3.2. Dose–Response Relationship of Eps

**Figure 2:**
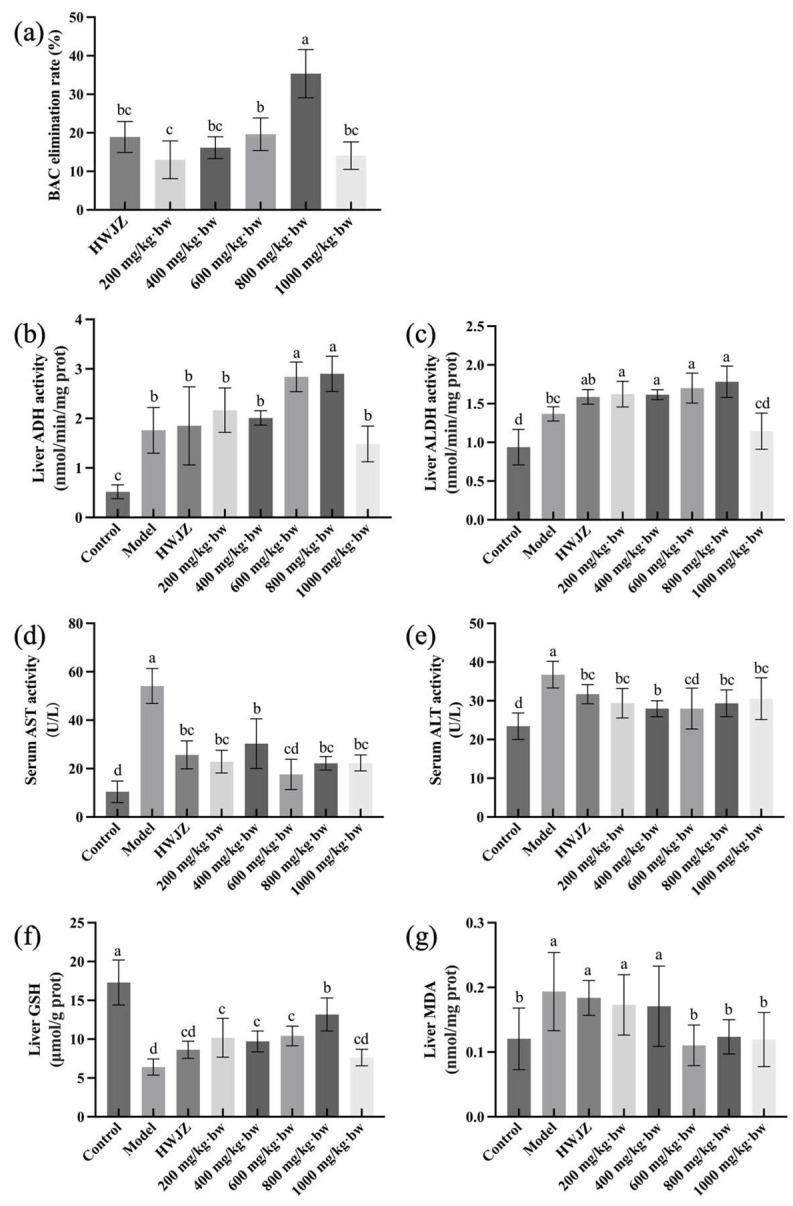
The effect of HWJZ and different doses of EPs on the BAC elimination rate (**a**), liver ADH (**b**) and ALDH (**c**) activity, serum AST (**d**), and ALT (**e**) activity, liver GSH (**f**) and MDA (**g**). Different letters indicate significant differences (*p* < 0.05).

As shown in Figure 2a, the BAC elimination rate of the positive control HWJZ was 18.93%. As the dose increased, the BAC elimination rate improved, reaching the maximum of 35.35% at the dose of 800 mg/kg·bw, which was significantly higher than in the other groups (*p* < 0.05). At this time, the active of EPs had already reached the peak of its effect, and no additional significant effect was obtained when the dose was further increased.

It has been shown that individual differences in ADH and ALDH indicate different individual tolerance to alcohol, which determines the speed of alcohol metabolism [8,37]. Thus, ADH and ALDH activities are critical for alcohol metabolism in humans. As shown in Figure 2b,c, both ADH and ALDH activities in the liver were significantly higher after the alcohol treatment than the normal group. With the gradual increase of EPs, the trends of ADH and ALDH activities were similar. The peaks were reached at 800 mg/kg·bw, 3.02 nmol/min/mg prot and 1.78 nmol/min/mg prot, respectively, which was consistent with the results of the BAC elimination rate. This suggests that EPs have higher activity in facilitating alcohol metabolism at 800 mg/kg bw.

When liver cells are damaged, the cell membranes’ permeability is increased, allowing transaminases to pass through the cell membranes and enter the blood circulation system [38,39]. Therefore, serum AST and ALT are often used as a sensitive indicator of the degree of early liver injury [40]. As shown in Figure 2d,e, compared with the normal group, the ALT and AST levels increased significantly after alcohol treatment (*p* < 0.05), indicating that liver cells were damaged. Importantly, serum ALT and AST levels decreased after EPs treatment and were minimized at 400 mg/kg·bw and 600 mg/kg·bw, respectively.

Alcohol metabolism generates large amounts of reactive oxygen species (ROS), and a large accumulation of ROS in the liver will cause oxidative stress, which is an important factor in alcoholic liver injury [21]. An antioxidant defense system exists in normal hepatocytes, including antioxidant enzymes (SOD, CAT, and GSH-Px, etc.) and non-enzymatic antioxidants (GSH, etc.), which can scavenge free radicals in the cellular environment and maintain the dynamic balance between ROS and antioxidant enzymes, thus protecting hepatocytes [13]. As shown in Figure 2f, compared with the normal group, GSH level was significantly decreased (*p* < 0.05) to 6.41 μmol/g prot in the model group after alcohol treatment. After positive control (HWJZ) treatment, the GSH level increased, but was not significantly different compared with the model group (*p* > 0.05). There was an Increase after different dosages of EPs treatment, and the 800 mg/kg·bw ePs group was the highest at 13.17 μmol/g prot, reaching 76.1% of the normal group’s value.

In humans, ROS act on lipids to cause peroxidation and the end product is malondialdehyde (MDA). Therefore, hepatic oxidative levels can be indicated by MDA levels [9,15]. As shown in Figure 2g, the MDA level in the model group increased to 157.1% (*p* < 0.05) of the normal group and decreased after 200 mg/kg·bw and 400 mg/kg·bw EPs treatments. After the 600 mg/kg·bw EPs treatment, the MDA decreased to the lowest level. As the dosage continued to increase, the MDA level did not decrease further (*p* > 0.05). Additionally, the BAC elimination rate and biochemical parameters of the EPs showed a clear dose–response relationship, and 800 mg/kg·bw EPs had the highest activity.

### 3.3. Amino Acid Analysis

As shown in Table 1, EPs contains a variety of amino acids and are rich in EAAs, which account for 42.45% of the TAAs. This has to do with the fact that eggs contain all EAAs [41]. Some studies have shown that Leu can significantly increase hepatic ADH activity [42,43]. Ala is involved in hepatic gluconeogenesis, which oxidizes NADH + H^+^ to NAD^+^, thereby maintaining cytoplasmic NAD^+^ concentrations and promoting alcohol metabolism [44]. The content of Leu and Ala in EPs were 5.26% and 3.25% respectively, accounting for 9.24% and 5.71% of the TAA. It is reported that HAA (Tyr, Trp, Gln, Val, Leu, Ile, Pro, Ala) exhibit the ability to facilitate alcohol metabolism in vivo [45]. According to the results of the test, EPs contains seven HAAs, with a content of 22.46%, accounting for 39.48% of all amino acids. The amino acid composition indicates that EPs has the material basis and potential effect of activating ADH and accelerating the rate of alcohol metabolism in vivo.

### 3.4. Identification of EPs by LC-MS/MS

**Figure 3:**
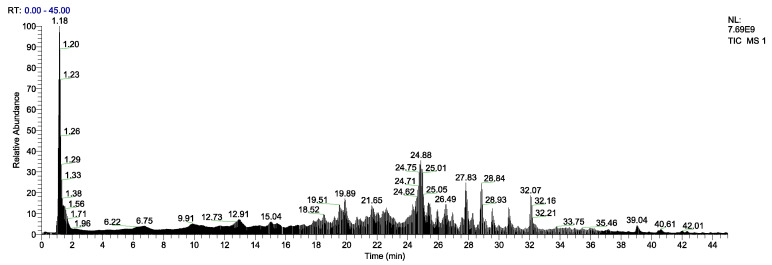
Total ion flow diagram of EPs.

The total ion flow diagram of EPs is shown in Figure 3. The retention time was mainly concentrated in the first 40 min. The obtained mass spectrometry peak data were compared with the egg protein database in Uniport (http://www.uniprot.org/ (accessed on 10 November 2023)) and a total of 84 peptides were obtained. As shown in Table 2, 84 peptide sequences from EPs were highly consistent with an egg source, by comparison with the online mass spectrometry databases provided by UniProt, NCBI and Ensembl [46,47]. The peptides sequences are mainly tetrapeptides and pentapeptides, and the MWs are mainly concentrated in 500–700 Da. Some studies have shown that peptides rich in HAA are more likely to cross the lipid barrier of intestinal mucosal cells to bind fatty acid radicals [44]. The identified peptides sequences were rich in HAA, especially Val, Leu, Ile, and Ala. They were also rich in antioxidant characteristic amino acids such as Leu and Pro. Pro can resist the ROS produced during alcohol metabolism, inhibit cell oxidation and release of inflammatory factors, slow down the excessive oxidation of lipid on the surface of liver cells, and play a hepatoprotective effect [48,49]. These amino acid compositions and sequence characteristics are an important structural basis for the EPs to exert their positive activities.

### 3.5. Molecular Docking and Visual Analysis

Alcohol dehydrogenase (ADH), a Zn containing metalloenzyme, is found in large quantities in the livers of humans and non-human animals, and in the cells of plants and microorganisms, and plays an important role in the metabolism of short-chain alcohols in organisms [36]. When a large amount of alcohol is ingested in a short period of time, many reactive oxygen species (ROS) will be produced, which will inhibit the activity of ADH and affect the rate of alcohol metabolism in the body [17,50]. Therefore, stabilizing or increasing the activity of ADH in organisms is an important strategy to promote alcohol metabolism. Molecular docking is an important modeling technique for predicting the major binding modes of receptor proteins to ligands and determining their binding energy [51]. In recent years, it has been widely used for the screening of biologically active proteins and small-molecule peptides, and a recent study using molecular docking to screen myeloperoxidase (MPO) inhibitors with antioxidant activity from abalone protein hydrolysate and sea cucumber protein hydrolysate has confirmed the accuracy and effectiveness of molecular docking [52,53]. The 84 peptides identified after docking with ADH are listed in Table 3 according to binding energy, from lowest to highest. As shown in Table 3, smaller MW tetrapeptides and pentapeptides had better docking results. Generally, if the binding energy of the ligand to the receptor protein is less than 0, it indicates that they can bind spontaneously, and if the binding energy is less than −6.0 kcal/mol, it indicates that the active ingredient has a strong affinity activity with the target [54,55]. Eighty-two of the peptides had binding energies of less than 0, and 13 had less than −6 kcal/mol, suggesting that the peptides contained in the EPs are capable of strong interaction with ADH, which may be closely related to their ability to facilitate alcohol metabolism. The four peptides that bound best were WIVD (−7.16 kcal/mol), IDNWE (−6.77 kcal/mol), KPIE (−6.76 kcal/mol), and TPVVD (−6.76 kcal/mol).

**Figure 4:**
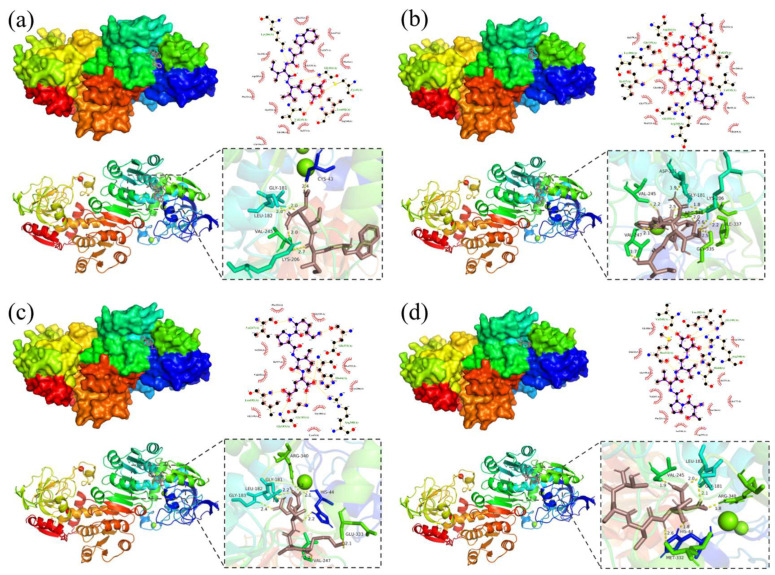
Docking results visualization of the four lowest binding energy peptides with ADH. WIVD (**a**), IDNWE (**b**), KPIE (**c**), and TPVVD (**d**).

As shown in Figure 4a, WIVD successfully docked into the hydrophobic cavity near the active center of ADH with the lowest binding energy of −7.16 kcal/mol. The cavity is mainly composed of HAA residues such as Cys-43, Leu-182, Val-247, and Met-270, and the other end is connected to the Zn of the active center. WIVD formed five hydrogen bonds with amino acid residues around the ADH active center, including Cys-43 and Leu-182, and produced hydrophobic interactions with many surrounding amino acids.

IDNWE visualization analysis is shown in Figure 4b. IDNWE formed a total of 10 hydrogen bonding interactions with eight amino acid residues near the ADH active center. In addition to hydrogen bonding interactions, it also formed strong hydrophobic interactions with several amino acid residues, such as Cys-43 and His-44.

As shown in Figure 4c, the result of KPIE with ADH, with a binding energy of −6.76 kcal/mol, was linked to seven amino acid residues, including residues Leu-182, Gly-181, and Gly-183, through hydrogen bonding, and hydrophobic interactions were formed. A previous study showed that the chicken-derived tripeptide KPC enhanced the stability of ethanol dehydrogenase through peptidase interactions and thus promoted alcohol metabolism [36].

As shown in Figure 4d, TPVVD successfully docked into the hydrophobic cavity near the ADH active center with a binding energy of −6.76 kcal/mol and was connected to the substrate amino acid residues through six hydrogen bonds, including Leu-182, Val-245, and Met-332 of the ADH active center. In addition to the hydrogen bonding interaction, many hydrophobic interactions are generated, which enhance the force of their action.

Overall, more than 95% of the peptides spontaneously bound to ADH, and 15.8% of the peptides had a high affinity to ADH, therefore EPs has the potential efficacy of promoting alcohol metabolism in terms of activating ethanol dehydrogenase, although the role played by amino acid fragments remains unclear.

### 3.6. In Vitro Activity Validation of Peptides

**Figure 5:**
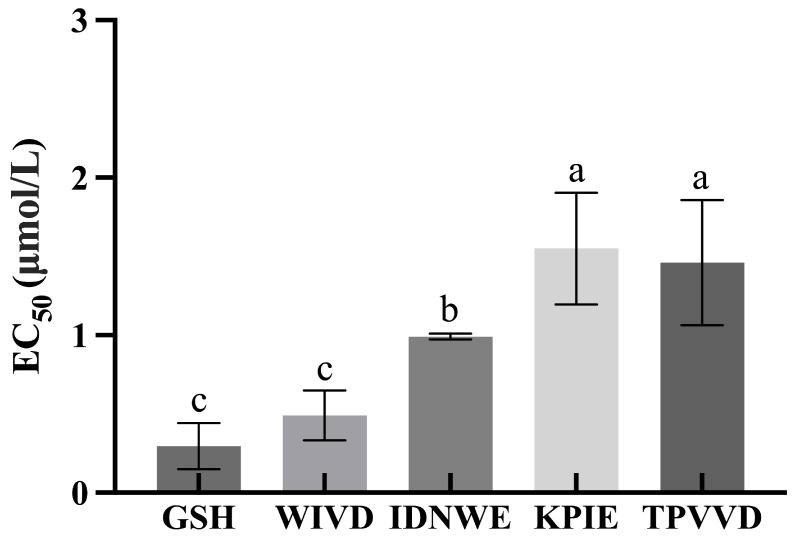
The EC_50_ values of ADH activation rates in vitro of different peptides. Different letters indicate significant differences (*p* < 0.05).

Based on the screening results of molecular docking, four peptides, WIVD, IDNWE, KPIE, and TPVVD, were selected and synthesized to evaluate their ADH activation rates in vitro, while glutathione (GSH) was selected as a positive control. GSH, a tripeptide composed of Glu, Cys, and Gly, is widely found in normal human cells, with the highest concentration in the liver. It plays an important role in scavenging free radicals, inhibiting inflammatory factors, relieving hangovers, and protecting the liver [56,57]. As shown in Figure 5, EC_50_ values of ADH activation rates in vitro for five peptides were obtained. EC_50_, also known as half effect concentration, is usually the concentration at which an exogenous agent causes a 50% change in a biological effect in the body [58]. The EC_50_ values of WIVD, IDNWE, KPIE, TPVVD, and GSH were calculated as 0.49 μM, 0.99 μM, 1.6 μM, 1.5 μM, and 0.30 μM, respectively. The EC_50_ value of GSH was the lowest, which is closely related to its involvement in alcohol metabolism and its hepatoprotective effects. Among the four peptides screened, the EC_50_ value of WIVD was much lower than that of the other three peptides and lower than that of the pentapeptide ILPHF screened by Zan et al., 2023, with EC_50_ value of 1.6 μM [59]. Moreover, in contrast, there was no significant difference between the EC_50_ values of GSH and WIVD (*p* < 0.05). These results indicated that WIVD was able to achieve the 50% effect at much lower concentrations, which is consistent with the bioactivity prediction as well as the molecular docking results. Based on the results of this experiment, WIVD has great potential as a peptide with facilitating alcohol metabolism.

## 4. Conclusions

The peptides with facilitating alcohol metabolism activity were prepared using optimal conditions and showed an obvious dose–response relationship in facilitating alcohol metabolism. Their alcohol metabolizing activity may be related to low MW and HAAs in the sequence, which have the potential to activate ADH activity. At the same time, it may be related to the antioxidant amino acid sequence, which can resist ROS produced by alcohol metabolism. Based on the 84 peptides identified, four peptides with the highest potential sobering activity were screened using molecular docking and validated in vitro. Among these, WIVD had the lowest EC_50_ value of 0.07307 μM. In summary, based on the results of our study and previous studies on biomolecules with alcohol metabolism promoting activity, EPs has potential as a functional food in facilitating alcohol metabolism. In the future, the structure–activity relationship of EPs will be further studied.

## Figures and Tables

**Table 1:** Amino acid compositions of the EPs.

Amino Acid	g/100 g of Sample
essential amino acid	
Ile *	2.94 ± 0.09
Leu *	5.26 ± 0.16
Lys	4.31 ± 0.13
Met *	1.88 ± 0.07
Val *	3.78 ± 0.11
Thr	2.74 ± 0.08
His	1.34 ± 0.04
Phe *	3.24 ± 0.11
Trp ^n.d.^	
non-essential amino acid	
Asx	6.06 ± 0.19
Glx	7.75 ± 0.27
Ser	4.31 ± 0.13
Gly	2.04 ± 0.06
Ala *	3.25 ± 0.09
Tyr	2.21 ± 0.08
Pro *	2.11 ± 0.07
Arg	3.67 ± 0.13
Cys ^n.d.^	
TAA	56.85 ± 1.82
EAA	25.46 ± 0.80
HAA	22.43 ± 0.71
EAA/TAA	44.78% ± 0.03%
HAA/TAA	39.44% ± 0.02%

Values are means (±SD) of triplicate analyses (*n* = 3). Three letter code have been used: Ile, L-isoleucine; Leu, L-leucine; Lys, L-lysine; Met, L-methionine; Val, L-valine; Thr, L-threonine; His, L-histidine; Phe, L-phenylalanine; Trp, L-tryptophane; Asx, L-asparagine + L-aspartic acid; Glx, L-glutamine + L-glutamic acid; Ser, L-serine; Gly, L-glycine; Ala, L-alanine; Tyr, L-tyrosine; Pro, L-proline; Arg, L-argnine; Cys, L-cysteine. * Hydrophobic amino acid (HAA). Trp n.d.: Not determined. Trp is degraded during acid hydrolysis. Cys ^n.d.^: Not determined. Cys is oxidized to cysteic acid under hydrolysis conditions. TAA: Total amino acid. EAA: Essential amino acid. n.d.: Not determined.

**Table 2:** Summary of peptides analyzed by LC-MS/MS.

Annotated Sequence	Top Apex RT [min]	Theo. MH+ [Da]
KDALN	1.2	560
TASEE	1.2	550
VSSEE	1.2	550
VSSDE	1.2	550
VLQAACRQE	1.2	1000
VNMDE	1.2	620
QEIE	1.2	520
QELD	1.2	520
QDIE	1.2	520
QDID	1.2	520
NEIE	1.2	520
CTASE	1.2	520
VGADD	1.5	490
LAAD	1.8	400
IAAE	1.8	400
AGDLD	5.3	520
GADLE	5.3	520
GADID	5.3	520
AGDIE	5.3	520
VAVE	5.4	420
VAVD	5.4	420
LGVE	5.4	420
IGVD	5.4	420
VSVE	7.4	430
VSVD	7.4	430
SSAME	7.4	520
SSAMD	7.4	520
IINE	12	490
IIND	12	490
IIGGD	12	490
SVGLF	14	500
VSGLE	14	500
VSGLD	14	500
ATGIE	14	500
KPKIE	16	630
KNNLE	16	620
SVGEVE	17	630
ILGEE	17	560
ILGED	17	560
ILGDE	17	560
IIGDD	17	560
SVDADID	18	730
IENL	18	490
VAALD	18	500
VAALE	18	500
VQIE	18	500
GIGLE	18	500
GIGID	18	500
KPID	18	500
AIGIE	18	500
ALGLD	18	500
KPIE	18	500
IAGLE	18	500
LAGLD	18	500
QVSELE	19	700
VSNELE	19	700
GVETDID	20	760
LSEDGIE	20	760
LDASDIE	20	760
VLQIE	22	600
VLNLE	22	600
VIAGIE	22	600
SVDIIE	22	680
VSDLLD	22	680
ATDLIE	22	680
QLGI	22	430
IIIDE	23	600
LIIDD	23	600
DLNWE	24	700
IDNWE	24	700
VAATL	25	490
WIVD	25	550
WIVE	25	550
VDVVD	25	560
TPVVE	26	570
TPVVD	26	570
TVIID	26	570
TVILE	26	570
LSLID	26	570
DIITVD	26	690
AVGGLVD	26	640
ISGDVL	26	620
IGVDVE	27	650
ASTIINME	29	900

**Table 3:** Molecular docking results.

Sequence	Binding Energy (kcal/mol)	Sequence	Binding Energy (kcal/mol)
WIVD	−7.16	QDID	−4.29
IDNWE	−6.77	IIGGD	−4.25
KPIE	−6.76	IENL	−4.25
TPVVD	−6.76	VDVVD	−4.22
LAAD	−6.72	ALGLD	−4.18
VAVE	−6.69	GADID	−4.17
WIVE	−6.61	SVDIIE	−4.13
QELD	−6.52	AGDIE	−4.10
VAATL	−6.46	CTASE	−4.06
ATGIE	−6.41	VLQIE	−4.06
VIAGIE	−6.23	AVGGLVD	−3.96
LGVE	−6.16	NEIE	−3.93
LIIDD	−6.03	IIGDD	−3.93
SVGLF	−5.84	TASEE	−3.91
TVIID	−5.83	KDALN	−3.85
QLGI	−5.75	LDASDIE	−3.85
IIIDE	−5.71	ILGDE	−3.79
IIND	−5.56	VSGLE	−3.69
KPID	−5.41	SSAME	−3.58
AIGIE	−5.30	SSAMD	−3.58
IAAE	−5.24	IINE	−3.53
LAGLD	−5.21	ILGEE	−3.53
VAVD	−5.19	VSGLD	−3.43
LSLID	−5.10	GADLE	−3.40
VSDLLD	−5.07	QVSELE	−3.36
VSVE	−5.06	VNMDE	−3.33
IGVD	−5.01	IGVDVE	−3.33
QEIE	−5.00	SVGEVE	−3.07
VSVD	−5.00	IAGLE	−3.02
VQIE	−4.94	VLNLE	−2.97
VAALD	−4.73	AGDLD	−2.94
VSSDE	−4.66	ILGED	−2.89
VAALE	−4.63	VSNELE	−2.57
TVILE	−4.57	DIITVD	−2.08
TPVVE	−4.52	DLNWE	−1.94
ATDLIE	−4.51	KNNLE	−1.78
ISGDVL	−4.48	SVDADID	−1.65
KPKIE	−4.43	GVETDID	−1.31
GIGID	−4.41	VSSEE	−1.25
VGADD	−4.38	LSEDGIE	−0.24
GIGLE	−4.38	VLQAACRQE	-
QDIE	−4.37	ASTIINME	-

## Data Availability

The data used during the current study are available from the corresponding author.
